# Kompromittierung durch additive Cerclagen: Ist der N. radialis in der operativen Versorgung der Humerusschaftfraktur in Gefahr?

**DOI:** 10.1007/s00113-021-00995-8

**Published:** 2021-03-24

**Authors:** F. von der Helm, J. Reuter, L. Adolf-Lisitano, E. Mayr, S. Förch

**Affiliations:** Klinik für Unfallchirurgie, Orthopädie, Plastische und Handchirurgie, Universitätsklinikum Augsburg, Stenglinstr. 2, 86156 Augsburg, Deutschland

**Keywords:** Humerusschaft, Cerclagen, Anterograde Nagelosteosynthese des Humerus, Primäre Radialisläsion, Sekundäre Radialisäsion, Humeral shaft, Cerclage, Anterograde nail osteosynthesis of the humerus, Primary radial nerve lesion, Secondary radial nerve lesion

## Abstract

**Einleitung:**

Die Therapie der Humerusschaftfraktur ist vielfältig und oft problematisch. Neben der konservativen Therapie konkurrieren Marknagel- und Plattenosteosynthese miteinander; bislang existiert kein Goldstandard. Aus biomechanischen Überlegungen bieten sich für die Versorgung von Spiralfrakturen additive Cerclagen an. Die Argumente gegen deren Verwendung sind zum einen die Gefahr von Radialisläsionen, zum anderen eine mutmaßliche Störung der Fragmentdurchblutung. Ziel dieser  Studie ist die Analyse sekundärer Radialisläsionen bei der Anwendung additiver, limitiert invasiver Cerclagen bei der antegraden Nagelosteosynthese von Humerusschaftfrakturen.

**Methodik:**

In dieser retrospektiven Studie erfolgen die klinische und die neurologische Untersuchung von 102 Patienten, welche im Zeitraum von 5 Jahren bei einer Humerusschaftfraktur operativ versorgt wurden. Insgesamt wurden zur Marknagelosteosynthese 193 Cerclagen durch einen limitiert invasiven Zugang eingebracht.

**Ergebnisse und Schlussfolgerung:**

Bei 4 Patienten (3,9 %) zeigte sich eine sekundäre Radialisläsion im Rahmen der operativen Stabilisierung. Die neurophysiologische und neurosonographische Untersuchung zeigten in keinem Fall eine Kompromittierung des Nerven durch Einschlingen oder gar Durchtrennung durch die additive Cerclage. Zwei Nervenläsionen erholten sich innerhalb von 3 bzw. 6 Monaten spontan. In den anderen 2 Fällen konnte der Verlauf aufgrund eines Exitus letalis nicht über 12 Monate dokumentiert werden.

Mit 3,9 % der iatrogenen Radialisläsionen liegt die Rate an Nervenläsionen im unteren Bereich dessen, was in der Literatur für die operative Therapie von Humerusschaftfrakturen beschrieben wird (3–12 %). Durch die limitiert invasive, additive Cerclage ergibt sich somit kein erhöhtes Risiko für die iatrogene Schädigung des N. radialis.

## Abstract (en)

**Background:**

In many cases the treatment of humeral shaft fractures is challenging and despite the large diversity of available approaches, no standard treatment exists. In addition to conservative treatment, intramedullary nails and plate osteosynthesis are competing methods for healing humeral shaft fractures. Furthermore, cerclage is considered to be an additive treatment for spiral fractures; however, this also increases the risk of radial nerve neuropathy and is said to compromise the perfusion of bone fragments. The goal of this study was to investigate secondary radial nerve neuropathy using additive and limited invasive cerclages for nail osteosynthesis of humeral shaft fractures.

**Methods:**

In the present study a total of 102 patients with humeral shaft fractures were clinically and neurologically re-examined after having been treated with nail osteosynthesis and additive cerclage via a limited invasive access over the past 5 years. In total 193 cerclages with limited invasive access were inserted during this time period.

**Results and conclusion:**

Of the patients four (3.9%) showed a secondary radial neuropathy during operative stabilization. Neurophysiological and neurosonographic examinations revealed that this had not been caused by compromising, embedding or severance of the radial nerve due to the cerclage. Two out of these nerve lesions recovered spontaneously within 3 and 6 months, respectively. The other two cases could not be documented over a period of 12 months due to death of the patient.

With 3.9% of iatrogenic radial nerve lesions the rate of nerve lesions falls into the lower range of that which has previously been described in the literature for nerve lesions due to operative treatment of humeral shaft fractures (3–12%). We thus conclude that there is no increased risk for iatrogenic injury of the radial nerve using additive and limited invasive cerclage.

## Einleitung

Die Humerusschaftfraktur ist eine seltene Fraktur eines großen Röhrenknochens. Sie wird mit einer Häufigkeit von 1–3 % aller Frakturen beziffert [1] und zeigt eine bimodale Altersverteilung mit Häufigkeitsgipfeln zwischen dem 20. und 30. sowie jenseits des 60. Lebensjahrs [2–4]. Verkehrsunfälle und Stürze aus größerer Höhe führen über eine direkte Gewalteinwirkung v. a. zu Querfrakturen, kurzen Schrägfrakturen oder Mehrfragmentfrakturen [3]. Beim Sturz auf die Hand werden v. a. bei älteren Menschen Torsionsfrakturen beobachtet. Aufgrund des demografischen Wandels wird sich die Inzidenz dieser Frakturentität in Zukunft um ein Vielfaches erhöhen. Somit wird auch die Bedeutung des Managements dieser Frakturen zunehmen [4, 5]. Mancherorts ist die Humerusschaftfraktur nach wie vor die Domäne der konservativen Therapie [6]. In der operativen Versorgung erfüllen neu entwickelte Implantate zunehmend die Ansprüche des modernen Patienten und erlauben eine lastfreie, frühfunktionelle Nachbehandlung [7]. Marknagel- und Plattenosteosynthese konkurrieren miteinander und stellen hohe Anforderungen an Chirurg, Implantat und operatives Vorgehen [8]. Biomechanisch wird die Osteosynthese im Gegensatz zur unteren Extremität weniger durch axiale, sondern v. a. durch Rotationskräfte belastet. Für zusätzliche Stabilität bei Schrägfrakturen könnten additive Cerclagen sorgen, die in einer experimentellen Studie eine signifikante Steigerung insbesondere der Rotationsstabilität an einem Tibia-Modell gezeigt haben [9]. Bei Oberarmfrakturen wird deren Einsatz aufgrund des Risikos iatrogener Radialisläsionen in Fachkreisen kontrovers diskutiert. In unserem Haus werden Humerusschaftfrakturen regelhaft mittels antegrader Marknagelosteosynthese in Kombination mit limitiert invasiven, additiven Cerclagen versorgt. Im Folgenden haben wir untersucht, ob dieses operative Vorgehen zu einer erhöhten Rate von sekundären Radialisläsionen führt.

## Methodik

Ein positives Ethikvotum des Universitätsklinikum Augsburg wurde unter dem Zeichen 2019-36 eingeholt. Alle volljährigen Patienten, die im Zeitraum von 5 Jahren (Januar 2015 bis März 2020) durch Marknagelosteosynthese und limitiert invasive, additive Cerclagen bei einer Humerusschaftfraktur versorgt worden waren, wurden in die retrospektive Analyse eingeschlossen. Zu den Ausschlusskriterien zählten Alter unter 18 Jahre, Mehrfachverletzungen an der ipsilateralen Extremität bei Polytrauma, Querfrakturen (<30°) (AO 12-A3), Trümmerfrakturen (AO 12-C3) und Frakturen mit Beteiligung des distalen Oberarms, da sich diese nicht für eine Stabilisierung durch additive Cerclagen eignen.

**Figure 1:**
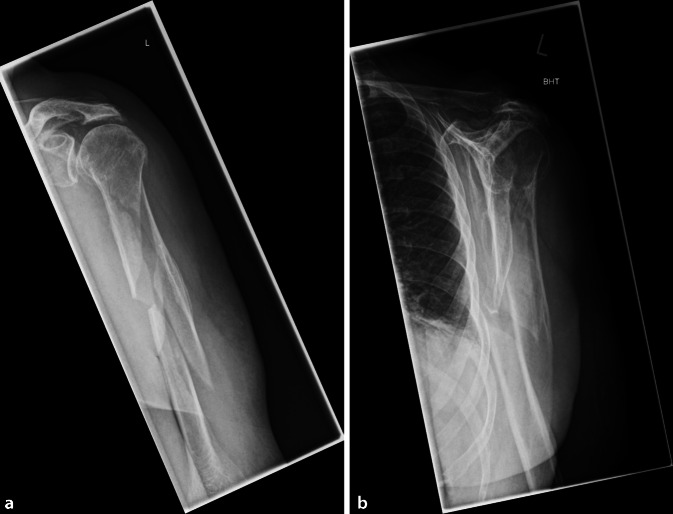

Über das Krankenhausdokumentationssystem wurden folgende Daten gewonnen: Alter, Geschlecht, Unfallmechanismus, Seite, Ausmaß der Weichteilverletzung, Begleiterkrankungen, Zeitpunkt der Operation, Schnitt-Naht-Zeit, Komplikationen und Liegedauer. Anhand der präoperativen radiologischen Bildgebung wurde die Frakturmorphologie nach AO/OTA (Arbeitsgemeinschaft für Osteosynthesefragen/Orthopaedic Trauma Association) klassifiziert (Abb. 1; [10]). Die Anzahl der verwendeten Cerclagen und deren Lokalisation in Bezug auf die Drittelgrenzen der Humeruslänge (absteigend von kranial) wurden dokumentiert. Sämtliche Komplikationen wurden erfasst und ausgewertet, diese Patienten wurden kontaktiert und nachuntersucht. Im Falle einer primären oder sekundären Radialisläsion erfolgte noch während des postoperativen primären stationären Aufenthaltes die Abklärung mittels fachneurologischer Konsiliaruntersuchung mit Neurosonographie und Messung der Nervenleitgeschwindigkeit. Im weiteren Verlauf notwendige Revisionsoperationen wurden ebenso erfasst wie auch Spätresiduen nach stattgehabter Verletzung des Nervus radialis.

## Operationstechnik

**Figure 2:**
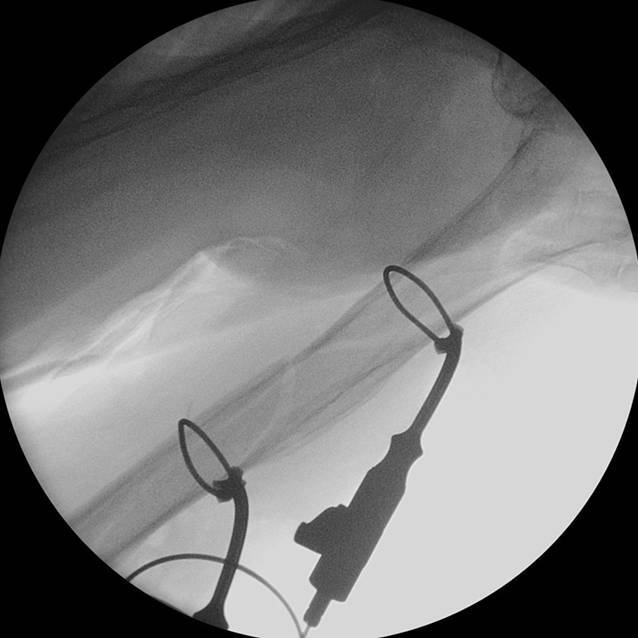

Die antegrade Marknagelosteosynthese mit additiver limitiert invasiver Draht-Cerclage führen wir standardmäßig in „Beach-chair“-Position mit Möglichkeit zur intraoperativen Durchleuchtung im a.-p. und im axialen Strahlengang durch. Zunächst wird die Frakturhöhe in der Durchleuchtung definiert und auf Höhe der geplanten Cerclage eine ca. 5 cm lange Hautinzision auf der Lateralseite des Oberarms angelegt. Nach vorsichtiger Durchtrennung der Faszie wird die Muskulatur mit dem Raspatorium sparsam vom Humerus abgeschoben, bis der Schaft mit dem Finger umfahren werden kann. Anschließend wird ein Umfahrer von dorsal eingesetzt. Eine in das Umfahrungsgerät eingelegte Redon-Drainage (Größe 8) wird vorgeschoben, sodass sie mit einer ventral am Humerus vorbeigeführten Overholt-Klemme gefasst und nach lateral aus der Wunde gezogen werden kann. Nun wird das Umfahrungsgerät entfernt und eine Draht-Cerclage (1,7 mm) in das freie Lumen der Drainage gesteckt. Das Herausziehen der weichen Redon-Drainage geht im Gegensatz zum starren Draht mit einer minimaler Weichteilkompromittierung einher. Hierüber kann die Cerclage mithilfe der Drainage eingezogen werden und umschlingt die Frakturfragmente des Humerus. Dank dieses Manövers über den Umweg der Redon-Drainage gelingt das Einbringen der Cerclage über den limitiert invasiven Zugang unter minimalem Ablösen der Weichteile vom Humerus. Die Integrität des N. radialis wird durch eine digitale Kontrolle geprüft. Durch axialen Zug und Rotation des distalen Fragmentes wird die Fraktur unter simultanem Anziehen der Cerclage anatomisch reponiert. Bevor die Cerclage zunächst manuell und anschließend mit dem Zuggerät angezogen wird, erfolgt eine weitere digitale Kontrolle des Nerven und der weiteren Leitungsbahnen. Bei Notwendigkeit weiterer Cerclagen wird das beschriebene Manöver entsprechend wiederholt (Abb. 2).

**Figure 3:**
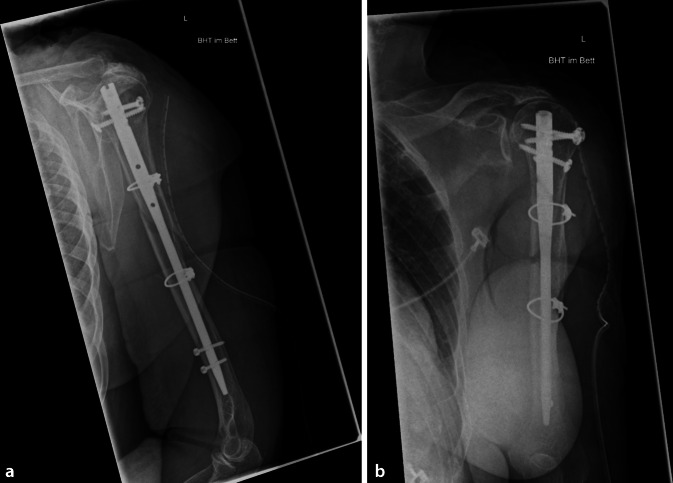

Es folgt nun nach präliminärer Stabilisierung die antegrade Verriegelungsnagelung des Humerus über einen Deltasplit-Zugang (Abb. 3).

## Ergebnisse

Es wurden insgesamt 102 Patienten aufgrund einer Humerusschaftfraktur mit einer antegraden Nagelosteosynthese und insgesamt 193 additiven Cerclagen operativ versorgt.

Das Durchschnittsalter lag bei 69 (28 bis 93) Jahren. Es waren 41 männliche und 57 weibliche Patienten. Bei 93 Patienten trat die Verletzung isoliert, bei 8 Patienten im Rahmen eines Polytraumas auf. Bei 22 Patienten war Alkohol die Sturzursache. In 53 Fällen zeigte sich die Frakturlokalisation rechts und in 49 Fällen links.

Die operative Versorgung erfolgte nach durchschnittlich 1,34 Tagen. Bei 72 Patienten erfolgte die operative Versorgung innerhalb 24 h. Die durchschnittliche Schnitt-Naht-Zeit lag bei 114 (65–204) min. Der Aufenthalt im Krankenhaus betrug im Schnitt 10 (2 bis 36) Tage.

**Figure 4:**
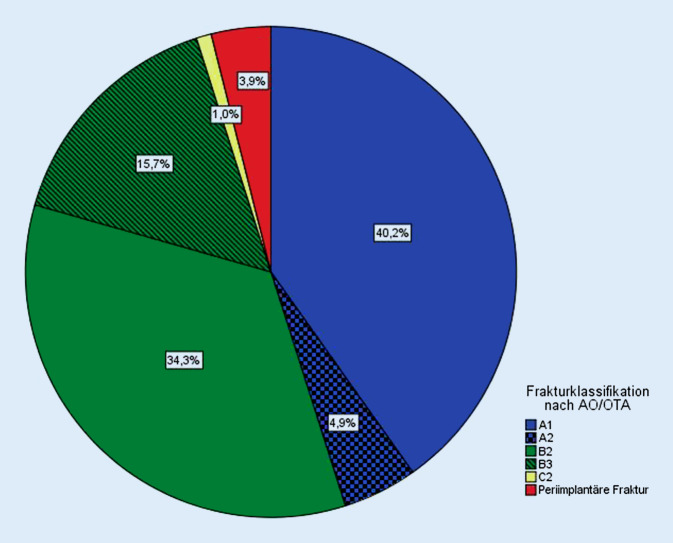

Entsprechend der Frakturklassifikation nach AO hatten 46 Patienten eine 12-A-, 51 eine 12-B- und 1 Patient eine 12-C-Fraktur. Eine periimplantäre Fraktur bestand in 4 Fällen (Abb. 4). Die Frakturlokalisation fand sich in 56 Fällen im mittleren und in 32 Fällen im proximalen Drittel des Humerusschaftes. In 2 Fällen befand sich die Fraktur im distalen Drittel. Zwölf Frakturen lagen genau auf Höhe des Übergangs zwischen dem proximalen und dem mittleren Drittel.

Insgesamt wurden in den 102 Operationen 193 additive Draht-Cerclagen um den Humerus eingebracht. 28 Frakturen wurden mit einer Cerclage und 58 Frakturen mit 2 Cerclagen durch einen limitiert invasiven Zugang versorgt. In 15 Fällen wurden *3* und in 1 Fall 4 Cerclagen eingebracht. Somit zeigt sich eine durchschnittliche Versorgung durch 1,89 (1 bis 4) Cerclagen je nach Frakturmorphologie.

**Figure 5:**
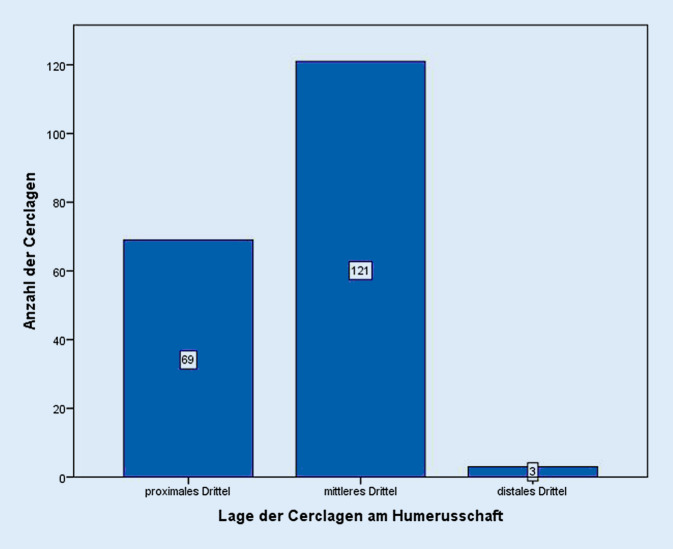

Die Lokalisation der Cerclagen bezogen auf die Dritteleinteilung am Humerusschaft zeigt sich am häufigsten mit 121 Cerclagen im mittleren Drittel. Im proximalen Drittel wurden 69 und im distalen Drittel 3 Cerclagen eingebracht (Abb. 5).

In insgesamt 14 Fällen (13,7 %) wurde eine Radialisläsion diagnostiziert. Diese lagen in 10 Fällen (9,8 %) schon posttraumatisch, also bereits präoperativ vor. In 4 Fällen (3,9 %) zeigte sich die Läsion erst postoperativ (Tab. 1).

**Table 1:** 

		Primäre Radialisläsion	Sekundäre Radialisläsion
*AO/OTA Klassifikation*	A1	3	2
	A2	2	–
	B2	4	1
	B3	1	1
*Anzahl der Cerclagen*	1	2	–
	2	6	2
	3	2	1
	4	–	1
*Lage der Cerclagen am Humerusschaft*	Mittleres Drittel	9	3
	Mehrere Drittel	1	1

*AO/OTA* Arbeitsgemeinschaft für Osteosynthesefragen/Orthopaedic Trauma Association

In allen Fällen konnte sowohl durch neurophysiologische als auch neurosonographische Untersuchungen eine direkte Schädigung oder ein Einschlingen des Nerven durch die Cerclagen ausgeschlossen werden. Die neurologischen Untersuchungen wiesen in allen Fällen auf einen Traktionsschaden hin. Damit ergibt sich eine Rate von 3,9 % iatrogener Radialisläsionen, bezogen auf die Anzahl an Operationen, und von 2,1 %, bezogen auf die Anzahl von eingebrachten Cerclagen.

## Diskussion

Die Therapie der Humerusschaftfraktur ist vielfältig und umstritten. Auch wenn sie derzeit nicht im Fokus der wissenschaftlichen Diskussion zu stehen scheint, macht es in unseren Augen Sinn, sich aufgrund der zu erwartenden demografisch begründeten Fallzahlsteigerungen mit dem Thema zu beschäftigen und eine Standardisierung zu entwickeln. Eine der häufigsten Komplikation ist eine Läsion des N. radialis [11]. Diese kann zu schweren funktionellen Ausfällen führen und den Patienten dauerhaft invalidisieren. Ein wesentlicher Faktor für die Entstehung ist der anatomische Verlauf des Nervens mit Durchtritt durch das Septum intermusculare laterale als weichteiliger Fixationspunkt am mittleren Oberarmdrittel.

In der aktuellen Literatur gibt es über die beste Operationstechnik keinen Konsens [3, 11]. Derzeit läuft die Diskussion hauptsächlich zwischen zwei Antipoden, einerseits der offenen Reposition und internen Fixation (ORIF) und andererseits der minimal-invasiven Marknagelung (IMN), seltener auch minimal-invasiv eingeschobener Plattenosteosynthese. Die Vorteile der ORIF sind die anatomische Reposition, die Darstellung des Nervens und die direkte interfragmentäre Kompression. Nachteilig ist die aufwendige chirurgische Präparation mit konsekutivem Weichteiltrauma und der Operationsdauer [7]. Die IMN beschreibt eine geringere Weichteilverletzung und bessere ästhetische Narben [3, 12]. Als Nachteile werden beim antegraden Nagel das Schulter-Impingement bei Materialüberstand und, daraus resultierend, die frühzeitig erforderliche Metallentfernung beschrieben [13]. In früheren Studien wird über eine postoperative Bursitis und Verletzungen der Rotatorenmanschette berichtet. Aufgrund der inzwischen verbesserten Operationstechnik zeigen sich alle diese Komplikationen deutlich seltener [7, 14]. Bezüglich eines komplikativen Verlaufs mit iatrogenen Nervenläsionen gibt es keine signifikanten Unterschiede [3, 7, 11, 13, 15, 16].

Die zumeist vorgebrachten Argumente gegen die Verwendung von Cerclagen sind die mögliche nachhaltige Kompromittierung der ossären Durchblutung und, damit verbunden, die Störung der Frakturheilung [17, 18]. Experimentelle Studien zur Kompromittierung der ossären Blutversorgung in der Fraktursituation existieren nicht. Am unverletzten Knochen konnte nur in einer Studie eine direkt postoperative Reduktion der Durchblutung nachgewiesen werden. Alle anderen Studien am unverletzten, osteotomierten oder im Wachstum befindlichen Knochen zeigten keine Beeinträchtigung der Blutversorgung, der Knochenheilung oder des Knochenwachstums durch Cerclagen [19].

Ein anderes Argument ist, dass die Cerclagen bereits nach kurzer Zeit auslockern und damit keine absolute Stabilität gewährleisten können. Dieser Effekt ist sicherlich richtig, spricht aber nicht zwingend gegen die Verwendung einer Cerclage als additives Vorgehen in Kombination mit einer relativ stabilen Osteosynthese wie einer Marknagelung oder einer Überbrückungsplatte. Wie Sandriesser und Förch in einer biomechanischen Studie [9] zeigen konnten, führt diese additive Verwendung einer Cerclage bei einer Überbrückungsplattenosteosynthese an der Tibia experimentell zu einer signifikanten Steigerung der Stabilität, sodass diese dem von Claes et al. als für die Frakturheilung optimal angesehenen Bereich nahekommt [20]. Durch diese zusätzliche Stabilität ist auch ein positiver Einfluss der Cerclage auf die Knochenheilung denkbar, und es scheint also durchaus legitim, sich darüber Gedanken zu machen, in welchen Bereichen eine additive Cerclage sinnvoll erscheint. Erste positive Ergebnisse dazu, insbesondere zur Reduzierung der Pseudarthroserate, liegen aus einer laufenden Studie vor.

Für den Einsatz am Humerus ist damit im Speziellen die Frage zu klären, ob mit einer additiven Cerclage das Risiko einer iatrogenen Radialisläsion steigt. In der vorliegenden deskriptiven Studie wird eine iatrogene Läsion des Nervens im Zusammenhang mit additiven Cerclagen in 4 Fällen beschrieben. Eine Vergleichsgruppe existiert nicht. Aufgrund der Fallzahl ist ein Vergleich mit der Literatur jedoch legitim. Schwab et al. berichteten von insgesamt 6 % (9 von 151 Fällen) sekundärer, iatrogener Läsionen nach Versorgung mit ORIF und IMN ohne Cerclagen [16]. In einer anderen Arbeit beschreiben Esmailiejah et al. nach konventioneller ORIF 4 von 33 (12 %) und nach minimal-invasiver Plattenosteosynthese (MIPO) eine von 32 (3 %) sekundäre Radialisläsionen [8]. Insgesamt wird in der Literatur, unabhängig von der Art der operativen Therapie (ORIF, MIPO, IMN), eine höhere Anzahl an sekundären Nervenschädigungen als in der vorliegenden Studie berichtet [6, 8, 15, 16, 21, 22]. Die hohe spontane vollständige Wiederherstellung der Nervenfunktion bei unseren Fällen korreliert dabei ebenfalls mit den Literaturdaten [11, 16] und ist ein weiterer Hinweis darauf, dass das hier beschriebene Vorgehen bei sorgfältiger Anwendung nicht den befürchteten Effekt für den Nerven hat. Die Operationsdauer mit einer durchschnittlichen Schnitt-Naht-Zeit von 114 (43–215) min zeigte im Literaturvergleich keine Auffälligkeiten und ist durch die Versorgung mit additiven Cerclagen nicht wesentlich verlängert [8]. Die neurologischen Schäden sind somit mutmaßlich der Traktion im Rahmen der Lagerung zuzuschreiben. Selbst wenn man dies außer Acht lässt und alle Schäden den Cerclagen zuschreibt, ergäbe sich eine Läsionsrate mit 2,1 %, welche immer noch niedriger ist, als für andere Operationsverfahren beschrieben.

## Schlussfolgerung

Insbesondere vor dem Hintergrund, dass in keinem einzigen Fall der von uns untersuchten Patienten neurophysiologisch oder neurosonographisch eine direkte Schädigung oder ein Einschlingen des Nerven durch die Cerclagen nachgewiesen werden konnte und die Gesamtrate an Radialisläsionen, bezogen auf die Anzahl der Frakturversorgungen, von 3,9 % im unteren Bereich der Literaturangaben liegt, scheint der Einsatz einer additiven, limitiert invasiven Cerclage bei der Versorgung von Humerusschaftfrakturen hinsichtlich der gefürchteten neurologischen Komplikationen keinesfalls obsolet zu sein. Bezieht man die nachgewiesenen postoperativen Radialisläsionen der vorliegenden Untersuchung auf die Anzahl der eingebrachten Cerclagen, ergibt sich eine Rate von 2,1 %. Ob sich die von uns erwarteten positiven Auswirkungen auf Frakturheilung, Revisionsrate und Rückführung der Patienten zum prätraumatischen Aktivitätslevel einstellen, ist Ziel einer weiteren in unserem Haus derzeit noch laufenden Untersuchung.

## Fazit für die Praxis

- Bei Humerusschaftfrakturen mit Spiral- oder Keilkomponenten sind Marknagelosteosynthesen mit additiven Cerclagen eine sichere Möglichkeit der operativen Versorgung. Komplikationen wie Nervenläsionen treten nicht vermehrt auf.
- Grundlegend für den Erfolg des Verfahrens ist das zielgenaue und gewebsschonende Einbringen der Cerclage vor der Implantation des Marknagels.
